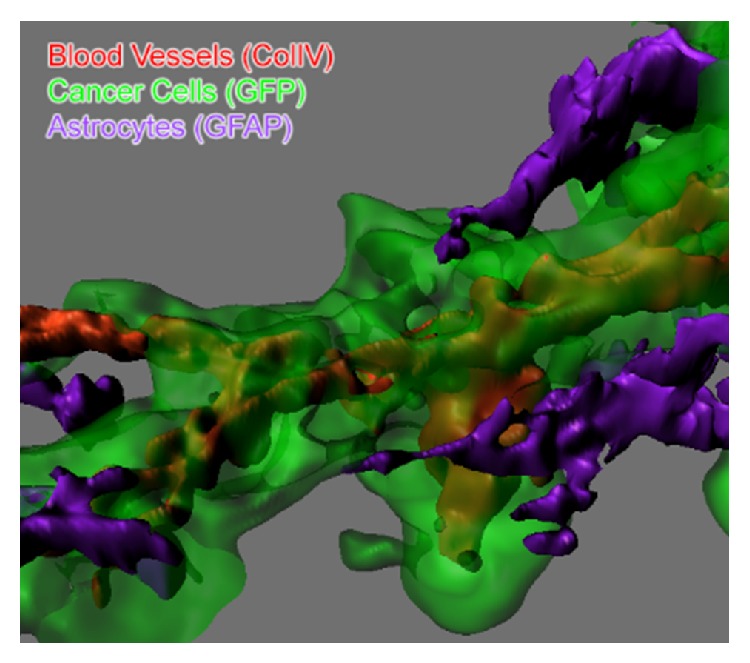# Understanding 3-Dimensional World from 2-Dimensional Immunofluorescent Adjacent Sections

**DOI:** 10.1155/2014/784937

**Published:** 2014-11-27

**Authors:** Sho Fujisawa, Dmitry Yarilin, Ning Fan, Mesruh Turkekul, Ke Xu, Afsar Barlas, Katia Manova-Todorova

**Affiliations:** Molecular Cytology Core Facility, Memorial Sloan-Kettering Cancer Center, New York City, NY 11235, USA

## Background

In many fields of biological sciences including embryology and cancer research, understanding of 3-dimensional structures is crucial to uncovering normal and pathological phenomena. While the most optimal method would be to directly observe the complete object without any destruction, staining and imaging of thick sections and whole mount samples can be challenging. For decades, researchers have serially sectioned large tissues stained each with chromogen-based immunohistological methods and painstakingly reconstructed the 3-dimensional volume. The limiting factor with immunohistological staining is the difficulty in detecting multiple antigens with different chromogens on the same tissue. At our Molecular Cytology Core Facility at Memorial Sloan-Kettering Cancer Center, we successfully and routinely perform immunofluorescent staining using automated staining machines and have combined IF staining and 3D reconstruction of serial sections. This method allows simultaneous detection of up to four different antigens on the same sections in a highly reproducible and specific manner. The resulting stack can be a stunning visualization of 3D structure and be quantitatively analyzed.

## Method

Human tumor samples as well as embryonic and adult mouse tissues were embedded in paraffin and sectioned serially. The slides were immunofluorescently stained for multiple antigens in sequential manner using Ventana Discovery XT (Ventana Medical Systems) autostainers. All slides were digitally scanned using Pannoramic FLASH (3DHistech) and autoaligned using Voloom (MicroDimensions) and/or AutoAligner (Bitplane). 3D visualization and analyses were performed using Imaris (Bitplane).

## Results

By combining automated immunofluorescent staining and tried-and-true method of reconstructing adjacent sections, we were able to visualize, in detail, not only the geometric structures of the sample but also the presence and interactions of multiple proteins and molecules of interest within their 3-dimensional environment (Figure 1).

## Conclusions

Advances in technology and software algorithms have significantly sped up the entire process of 3D reconstruction of serial sections. The addition of automated, multiantigen, immunofluorescent staining will significantly broaden the range and complexity of scientific questions that can be answered with this methodology.

## Figures and Tables

**Figure 1:**